# Crohn’s-associated invariant T cells (CAITs) recognise small sulfonate molecules on CD1d

**DOI:** 10.1136/gutjnl-2022-328684

**Published:** 2022-11-25

**Authors:** Anastasia A Minervina, Mikhail V Pogorelyy, Steffen Paysen, Ulrich Luening, Frauke Degenhardt, Andre Franke, Paul G Thomas, Elisa Rosati

**Affiliations:** 1 Department of Immunology, St Jude Children's Research Hospital, Memphis, Tennessee, USA; 2 Otto-Diels-Institute for Organic Chemistry, Christian-Albrecht University of Kiel, Kiel, Germany; 3 Institute of Clinical Molecular Biology, University Hospital Schleswig Holstein, Kiel, Germany; 4 Institute of Immunology, Christian-Albrecht University of Kiel, Kiel, Germany

**Keywords:** CROHN'S DISEASE, T LYMPHOCYTES, T-CELL RECEPTOR

## Main

In the recent study by Rosati *et al,* we described a novel unconventional T cell population enriched in the peripheral blood of patients with Crohn’s disease (CD) and characterised by a semi-invariant T cell receptor (TCR) repertoire.^1^ However, the specificity of these Crohn’s-associated invariant T (CAIT) cells was not defined. Identifying the specificity of CAIT cells is essential to understand the origin of the antigen triggering their enrichment in CD.

**Figure 1:**
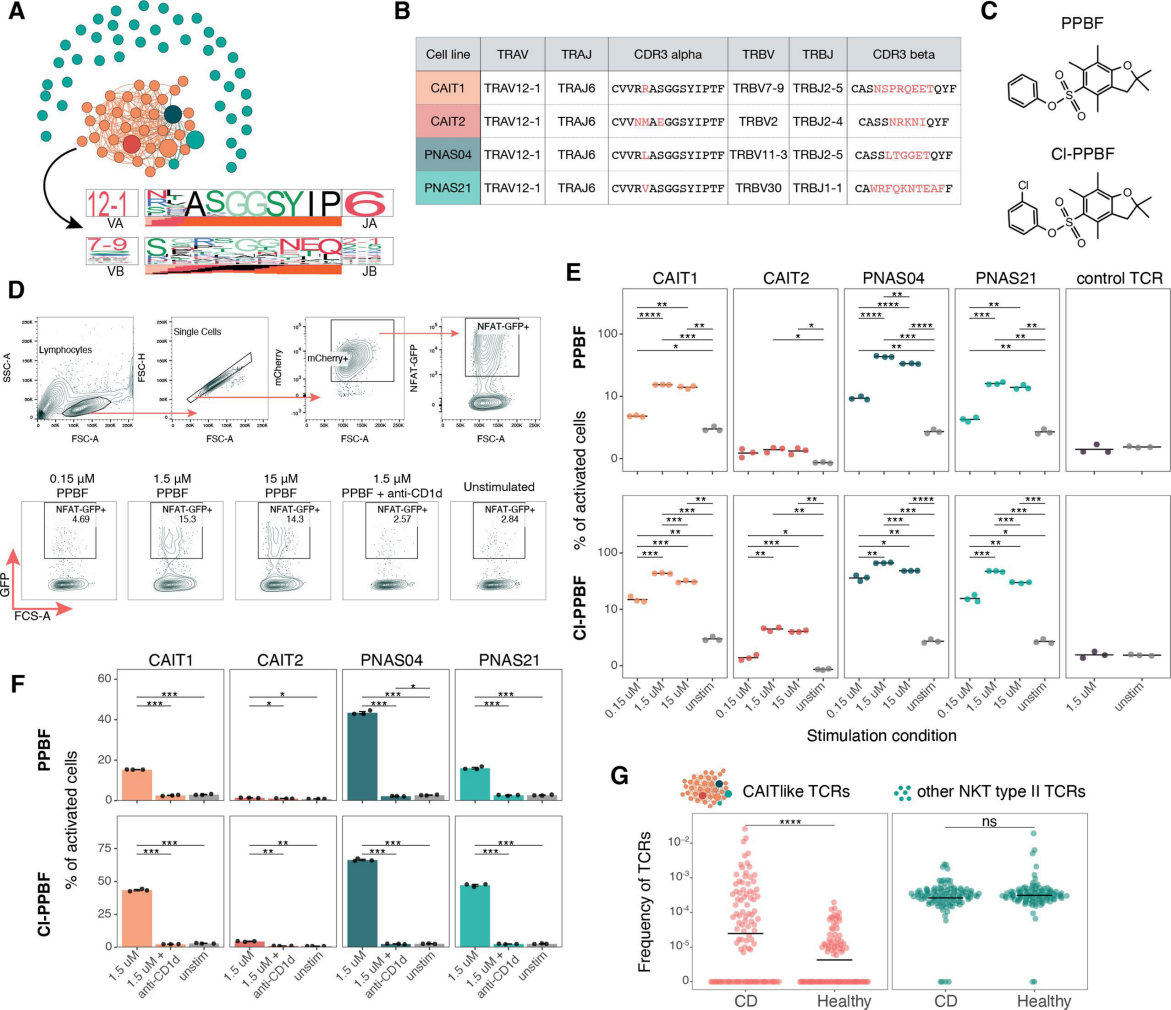
Comparison of natural killer T (NKT) type II and Crohn’s-associated invariant T (CAIT) cells. (A) Sequence similarity analysis of NKT type II^2 3^ and CAIT T cell receptors (TCRs). Each node corresponds to the unique alpha/beta TCR sequence and edges connect highly similar TCRs (tcrdist metric<150). Larger nodes indicate TCRs used for cloning. TCRdist sequence logos for TCRalpha and TCRbeta chains of TCRs from the cluster are shown at the bottom. (B). Genomic segments and amino acid CDR3 sequences of TCRs picked for experimental validation. Red font indicates differences in CDR3 regions. (C). Chemical structures of phenyl-pentamethyldihydrobenzofuransulfonate (PPBF) and chlorophenyil-pentamethyldihydrobenzofuransulfonate (ClPPBF). (D.) Gating strategy and representative flow plots for pentamethylbenzofuransulfonates (PBFs) stimulation experiment. (E). Frequency of activated cells reactive to PPBF (top) and ClPPBF (bottom). Only significant p values from a T-test with Holm method for multiple testing correction are shown (*<0.05, **<0.01, ***<0.001, ****<0.0001). (F). Anti-CD1d antibody prevents activation of all four cell lines with PPBF (top) and ClPPBF (bottom). Only significant p values from a t-test with Holm method for multiple testing correction are shown (*<0.05, **<0.01, ***<0.001, ****<0.0001). (G.) Frequency of CAITlike NKT type II TCRs from Almeida *et al* (attached to a large cluster on figure 1A) and non-CAIT like NKT type II TCRs in Crohn’s disease and healthy cohorts from Rosati *et al* .^1^ Only significant p value (****<0.0001) from a Mann-Whitney U-test is shown.

In our previous study, we observed that CAIT cells have TCRs similar to those reported for some natural killer T (NKT) type II cells.^2 3^ Here, we performed a sequence similarity analysis^4^ and identified a large cluster composed of CAIT clonotypes and three reported NKT type II clonotypes (figure 1A). While the NKT type II and CAIT clonotypes all had highly similar TCR alpha chains carrying TRAV12-1/TRAJ6 genomic segments, their beta chains were highly diverse (figure 1A, bottom). Dash *et al* have shown that TCRs with similar sequences frequently have the same specificity.^5 6^ In the original publications describing these clonotypes, the authors reported that the NKT type II cells recognise small molecules of the pentamethylbenzofuransulfonates (PBFs) family presented by the invariant HLA-like CD1d protein.^2 3^ Thus, we investigated whether CAIT TCRs shared the specificity of the NKT type II cells.

We transduced TCR-null NFAT-GFP reporter Jurkat cells with constructs encoding two representative CAIT TCRs and two coclustering NKT type II TCRs (figure 1B). We synthetised two of the PBF compounds, phenyl-pentamethyldihydrobenzofuransulfonate (PPBF), the original compound identified as a CD1d-dependent activator of NKT type II cells,^2^ and a more potent PPBF analogue chlorophenyil-pentamethyldihydrobenzofuransulfonate (ClPPBF)^3^ (figure 1C). Importantly, Jurkat cells naturally express CD1d and thus can act as antigen-presenting cells for CD1d-dependent antigens. To evaluate TCR activation, we cultured Jurkat cell lines with incremental concentrations of PPBF and ClPPBF (figure 1D).

All four transgenic cell lines (CAIT1, CAIT2, PNAS04, PNAS21) reacted to both compounds in a dose-dependent manner (figure 1E). Consistent with the original study, all tested TCRs reacted more strongly to ClPPBF. The CAIT2 cell line, with the lowest level of activation, (figure 1E) carried the most dissimilar TCRalpha sequence compared with the other cell lines. CAIT2 has mismatches at CDR3 positions 4 and 7 (figure 1B), suggesting the importance of these positions for TCR avidity. A control cell line with known TCR specificity was not activated by the compounds, indicating that compound recognition is TCR-dependent (figure 1E). The reactivity of all cell lines dropped to unstimulated levels in the presence of the CD1d-blocking antibody, demonstrating that the TCR interaction is CD1d-restricted (figure 1F).

To further investigate the possible role of NKT type II cells in CD, we searched all NKT type II sequences reported in Almeida *et al* in our previously published TCR data from patients with CD and healthy controls.^1 3^ Only TCRs from NKT type II cells with the characteristic CAIT TCRalpha chain motif showed significant enrichment in patients with CD, while other TCRs were found in comparable amounts in patients and controls (figure 1G). Thus, only a subgroup of NKT type II cells with specific TCR features is enriched in CD.

While we show the specificity of CAIT cells for PBF small molecules in the context of CD1d, many questions remain. Many other small molecules similar to PBFs exist, including drug derivatives and microbial metabolites.^3 7 8^ It is thus reasonable to hypothesise that different small molecules may be triggering CAIT cells in vivo. Importantly, NKT cells can differentiate into opposing phenotypes, from proinflammatory to regulatory,^8–10^ necessitating further characterisation on the functional profile of the NKT type II CAIT cell subset and its behaviour in patients with CD.

Detailed methodologies are described in online supplemental file 1.

10.1136/gutjnl-2022-328684.supp1 Supplementary data
