# Supplementary material for: Crohn’s-associated invariant T cells (CAITs) recognise small sulfonate molecules on CD1d
Source: Gut. 2022 Nov 25;73(1):205–6. doi: 10.1136/gutjnl-2022-328684 (PMC10715465; doi:10.1136/gutjnl-2022-328684)
Supplement: Supplementary data [file gutjnl-2022-328684supp001.pdf]

Crohn's associated invariant T cells (CAITs) recognize small sulfonate molecules on CD1d

Minervina, Pogorelyy *et al.*

SUPPLEMENTARY MATERIALS AND METHODS

Compound synthesis

General information

Chromatography was carried out with Isolera™ spectra system with ACI™ and assistant Fa. Biotage with automated fraction collector. The used columns were Biotage® SNAP Ultra cartridge filled with Biotage® HP-Sphere™ spherical silica. Reactions were monitored with a UV detector and the R_f-values were determined by TLC-plates (Alugram® Xtra Sil G/UV254 Fa. Machery-Nagel). All solvents were destilled before use.

NMR-Spectroscopy

In addition to the ^{1}H and ^{13}C NMR Spectra, two-dimensional measurement methods for further structural elucidation were measured (COSY, HSQC, HMBC). The following designations were used for the assignment of the signals:

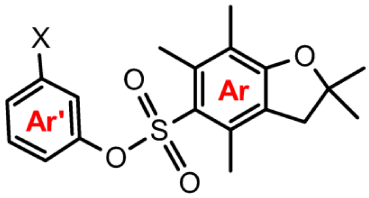

Chemicals

Chemical name	Purity	Company
chlorosulfuric acid	99 %	Fluka
3-chlorophenol	98 %	TCI
cyclohexane	99 %	Walter-CMP
dichloromethane	99 %	Walter-CMP
ethyl acetate	99 %	Walter-CMP
isobutyraldehyde	98 %	TCI
potassium carbonate	99 %	Grüssing
phenol	99.5 %	TCI
toluene	99 %	Walter-CMP
2,3,5-trimethylphenol	98 %	TCI
sulfuric acid	96 %	Grüssing

Crohn's associated invariant T cells (CAITs) recognize small sulfonate molecules on CD1d

Minervina, Pogorelyy *et al.*

Synthesis of 2,2,4,6,7-pentamethyl-2,3-dihydrobenzofuran (1)

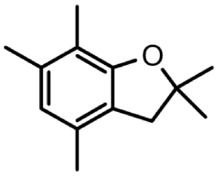

Isobutylaldehyde (78 mmol) and 300 µL of sulfuric acid were added to a stirred solution of 8.88 g of 2,3,5-trimethylphenol (65.3 mmol) in 50 mL of toluene. The reaction mixture was refluxed for 20 h. After cooling to room temperature, 200 mL of ethyl acetate and 50 mL of demineralized water were added. The organic layer was washed with saturated potassium carbonate solution (2 x 100 mL) and demineralized water (2 x 100 mL). The solvent was removed under reduced pressure. The residue was purified by chromatography (cyclohexane (R_f = 0.24) □ ethyl acetate/cyclohexane 1:9 (R_f = 0.47) to afford **1** as a white solid.

yield: 6.90 g (36.3 mmol, 56 %) Ref.: 76 % [1]

^{1}H-NMR (200 MHz, $CDCl_3$): δ = 6.48 (s, 1H, 5-Ar-*H*), 2.90 (s, 2H, CH_2), 2.19 (s, 3H, 4-Ar-CH_3), 2.14 (s, 3H, 6-Ar-CH_3), 2.07 (s, 3H, 7-Ar-CH_3), 1.46 (s, 6H, 2-Ar-CH_3) ppm.

5-(2,2,4,6,7-Pentamethyl-2,3-dihydrobenzofuran)-sulfonyl chloride (2)

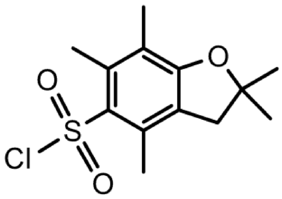

Under nitrogen, a solution of **1** (502 mg, 2.64 mmol) in 25 mL of dry dichloromethane was cooled to 0 °C. 0.35 mL of chlorosulfuric acid (5.26 mmol) was added dropwise and the reaction mixture was stirred for 3 h at room temperature. The crude product solution was used for the synthesis of **3** and **4** without further purification or analysis.

Crohn's associated invariant T cells (CAITs) recognize small sulfonate molecules on CD1d

Minervina, Pogorelyy *et al.*

Phenyl-5-(2,2,4,6,7-pentamethyl-2,3-dihydrobenzofuran)-sulfonate (3)

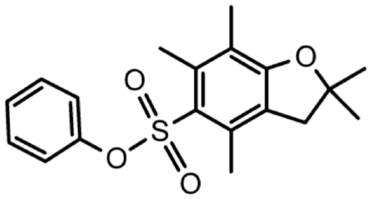

Under nitrogen, 735 mg of phenol (7.92 mmol) was stirred in 25 mL of dry dichloromethane at 0 °C. During 1 h, freshly prepared sulfonyl chloride **2** was added dropwise to the reaction. The reaction mixture was stirred for 20 h and quenched with 50 mL of demineralized water. Dichloromethane (200 mL) were added to the organic layer, and it was washed with a saturated potassium carbonate solution (1 x 100 mL) and demineralized water (2 x 100 mL). The solvent was removed under reduced pressure. The residue was purified by chromatography (cyclohexane (R_f = 0.11) □ ethyl acetate/cyclohexane 1:9 (R_f = 0.26) to afford **1** as a yellow oil.

yield.: 245 mg (707 µmol, 13 %) Ref.: 55 % [1]

^{1}H-NMR (600 MHz, $CDCl_3$): δ = 7.29 (t, ^3J = 7.4 Hz, 2H, 3-Ar'-*H*, 5-Ar'-*H*), 7.22 (t, ^3J = 7.4 Hz, 1H, 4-Ar'-*H*), 7.03 (d, ^3J = 7.4 Hz, 2H, 2-Ar'-*H*, 6-Ar'-*H*), 2.95 (s, 2H, CH_2), 2.56 (s, 3H, 4-CH_3), 2.33 (s, 3H, 6-CH_3), 2.13 (s, 3H, 7-CH_3), 1.46 (s, 6H, 2-CH_3) ppm.

^{13}C-NMR (150 MHz, $CDCl_3$): δ = 161.0 (s, 5-Ar-*C*), 149.6 (s, 1-Ar'-*C*), 141.2 (s, 7a-Ar-*C*), 135.1 (s, 7-Ar-*C*), 129.5 (d, 3-Ar'-*C*, 5-Ar'-*C*), 126.8 (d, 4-Ar'-*C*), 125.2 (s, 4-Ar-*C*), 125.1 (s, 6-Ar-*C*), 122.4 (d, 2-Ar'-*C*, 6-Ar'-*C*), 118.2 (s, 3a-Ar-*C*), 87.3 (s, 2-*C*), 42.8 (t, 3-*C*H_2), 28.5 (q, 2-*C*H_3), 19.2 (q, 6-Ar-*C*H_3), 17.8 (q, 4-Ar-*C*H_3), 12.5 (q, 7-Ar-*C*H_3) ppm.

^{1}H-NMR-purity ≥ 95 %

Crohn’s associated invariant T cells (CAITs) recognize small sulfonate molecules on CD1d

Minervina, Pogorelyy *et al.*

3-Chlorophenyl-5-(2,2,4,6,7-pentamethyl-2,3-dihydrobenzofuran)-sulfonate (4)

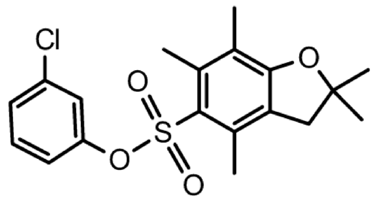

Under nitrogen, 1.47 g of 3-chlorophenol (11.5 mmol) was stirred in 25 mL of dry dichloromethane at 0 °C. During 1 h, freshly prepared sulfonyl chloride **2** was added dropwise to the reaction. The reaction mixture was stirred for 20 h and quenched with 50 mL of demineralized water. Dichloromethane (200 mL) were added to the organic layer, and it was washed with a saturated potassium carbonate solution (2 x 100 mL) and demineralized water (2 x 100 mL). The solvent was removed under reduced pressure. The residue was purified by chromatography (ethyl acetate/cyclohexane 9:1 (R_f = 0.11) □ 1:1 (R_f = 0.27) to afford **1** as a yellow oil.

yield.: 175 mg (460 µmol, 9 %) Ref.: 44 % [1]

^{1}H-NMR (500 MHz, $CDCl_3$): δ = 7.23 (m_c, 1H, 4-Ar'-*H*), 7.22 (d, ^4J = 1.2 Hz, 1H, 2-Ar'-*H*), 7.05 (m_c, 1H, 5-Ar'-*H*), 6.96 (m_c, 1H, 6-Ar'-*H*), 2.95 (s, 2H, 2-CH_2), 2.56 (s, 3H, 4-CH_3), 2.33 (s, 3H, 6-CH_3), 2.13 (s, 3H, 7-CH_3), 1.46 (s, 6H, 2-CH_3) ppm.

^{13}C-NMR (125 MHz, $CDCl_3$): δ = 161.0 (s, 1C, 5-Ar-*C*), 150.0 (s, 1C, 1-Ar'-*C*), 141.0 (s, 1C, 7a-Ar-*C*), 135.1 (s, 1C, 7-Ar-*C*), 134.6 (s, 1C, 3-Ar'-*C*), 130.2 (d, 1C, 4-Ar'-*C*), 127.4 (d, 1C, 2-Ar'-*C*), 125.4 (d, 1C, 4-Ar-*C*), 124.5 (s, 1C, 6-Ar-*C*), 122.9 (d, 1C, 5-Ar'-*C*), 120.7 (d, 1C, 6-Ar'-*C*), 118.5 (s, 1C, 3a-Ar-*C*), 87.5 (s, 1C, 2-*C*), 42.9 (t, 1C, 3-*C*H_2), 28.5 (q, 2C, 2-*C*H_3), 19.1 (q, 1C, 6-Ar-*C*H_3), 17.9 (q, 1C, 4-Ar-*C*H_3), 12.5 (q, 1C, 7-Ar-*C*H_3) ppm.

^{1}H-NMR-purity ≥ 90 %

Crohn's associated invariant T cells (CAITs) recognize small sulfonate molecules on CD1d

Minervina, Pogorelyy *et al.*

Jurkat Cell Line generation

Gene fragments encoding TCRalpha (modified with mouse TRAC), TCRbeta (modified with mouse TRBC), and mCherry construct connected with the T2A(TRA-TRB junction) and P2A linkers (TRB mCherry junction) were ordered from Genscript and cloned into pLVX-EF1a-IRES-Puro lentiviral expression vector (Clontech). 293T cells (ATCC CRL-3216) were transfected with the parental plasmid together with psPAX2 packaging plasmid (Addgene plasmid #12260), and pMD2.G envelope plasmid (Addgene plasmid #12259) using Lipofectamine 3000 kit (Invitrogen). Lentivirus-containing media was collected 24 and 48 hours post-transfection. Lenti-X Concentrator (Clontech) was used for lentivirus precipitation according to the manufacturer's protocol. TCR-null Jurkat cell line expressing endogenous NFAT-GFP reporter (gift from Wouter Scheper) was transduced with a lentivirus and antibiotic selected for 1 week using 1 mg/mL puromycin in RPMI (Gibco) containing 10% FBS and 1% penicillin/streptomycin. Transduction of Jurkat cell line was confirmed by expression of mCherry.

Transgenic cell lines stimulation and flow analysis

Jurkat cells were seeded into round-bottom 96-well plates at 100 000 cells per well. Cells were stimulated with serial dilution of PPBF (15uM, 1.5 uM, 0.15uM in DMSO) and ClPPBF compounds (15uM, 1.5 uM, 0.15uM in DMSO) in 100 ul of stimulation media, containing 1 mg/mL each of anti-human CD28 and CD49d (BD Biosciences). An unstimulated (CD28, CD49d) and positive control (CD28, CD49d, 1X Cell Stimulation Cocktail, PMA/ionomycin; eBioscience) were included in each assay. Each experimental condition was performed in triplicates. For the CD1d blocking experiment 5ul of anti-CD1d (clone 51.1, Biolegend) was added to each well. Transgenic Jurkat cell line expressing the TCR known to be specific for SARS-CoV-2 epitope was used as a negative control. TCR activation was assessed using NFAT-GFP expression after 18 h incubation (37 C, 5% CO_2) by flow cytometry on a BD Fortessa using FACSDiva software. Flow data were analyzed in FlowJo software.

Crohn's associated invariant T cells (CAITs) recognize small sulfonate molecules on CD1d

Minervina, Pogorelyy *et al.*

TCR sequence analysis

To identify a cluster of highly similar TCR sequences we used a TCRdist network approach as described in ref Cell reports Medicine. The pairwise TCRdist metric and motif logo for the largest CAIT-like cluster was generated with *conga*[1]. The TCR similarity network (with tcrdist<150 threshold for edges) was built with the igraph package and visualized using *gephi*.

All TCR alpha chains from published NKT type II cells reactive to the small sulfonate compounds[2 3] were searched in previously published bulk TCR repertoire dataset[4] including 109 Crohn's disease (CD) patients and 99 healthy controls. TCRs were looked for by the combination of their amino acid CDR3 sequence, their V gene and J gene. To identify whether NKT type II clonotypes were enriched in CD patients, we extracted from the reference dataset all TCR alpha sequences carrying the same VJ combination and differing by maximum one amino acid from any of the query TCRs. The so identified clonotypes were then grouped in two categories (i) CAIT-like clonotypes, carrying the TRAV12-1, TRAJ6 genes and motif CVV**A*GGSYIPTF as previously described[4] and (ii) other NKT type II clonotypes. The relative abundance of each clonotype in the respective category was summed thus obtaining the cumulative abundance of (i) CAIT-like and (ii) other NKT type II clonotypes in each individual.

Reference

1. Schattgen SA, Guion K, Crawford JC, et al. Integrating T cell receptor sequences and transcriptional profiles by clonotype neighbor graph analysis (CoNGA). *Nature Biotechnology* 2022;40(1):54-63. doi: 10.1038/s41587-021-00989-2
2. Almeida CF, Smith DGM, Cheng T-Y, et al. Benzofuran sulfonates and small self-lipid antigens activate type II NKT cells via CD1d. *Proceedings of the National Academy of Sciences* 2021;118(34):e2104420118. doi: 10.1073/pnas.2104420118
3. Van Rhijn I, Young DC, Im JS, et al. CD1d-restricted T cell activation by nonlipidic small molecules. *Proc Natl Acad Sci U S A* 2004;101(37):13578-83. doi: 10.1073/pnas.0402838101 [published Online First: 2004/09/03]
4. Rosati E, Rios Martini G, Pogorelyy MV, et al. A novel unconventional T cell population enriched in Crohn's disease. *Gut* 2022:gutjnl-2021-325373. doi: 10.1136/gutjnl-2021-325373
